# Multidisciplinary perspectives on quality improvement areas for consultation-liaison psychiatry services in Australian hospitals

**DOI:** 10.1177/10398562261424837

**Published:** 2026-02-23

**Authors:** Murray G. Tucker, Zachary Fitzgerald, Emma Nicholson, Julia Hunt, Steven Moylan

**Affiliations:** Mental Health, Drugs and Alcohol Services (MHDAS), 3487 Barwon Health, Geelong, VIC, Australia; School of Medicine, 2104 Deakin University, Geelong, VIC, Australia; Mental Health, Drugs and Alcohol Services (MHDAS), 3487 Barwon Health, Geelong, VIC, Australia; Mental Health, Drugs and Alcohol Services (MHDAS), 3487 Barwon Health, Geelong, VIC, Australia; School of Nursing, 2104 University of Tasmania, Hobart, TAS, Australia; School of Medicine, 2104 Deakin University, Geelong, VIC, Australia

**Keywords:** consultation-liaison psychiatry, multidisciplinary, quality improvement, staff satisfaction, referral and consultation

## Abstract

**Objective:**

This study investigated the perspectives of multidisciplinary staff regarding consultation-liaison psychiatry (CLP) services in Australian public hospitals.

**Method:**

A cross-sectional survey was distributed to CLP service providers (CLP staff and managers) and non-CLP hospital staff (allied health professionals, peer workers, and medical staff from Victoria and Tasmania). The survey assessed perceptions of response times, satisfaction, and areas for improvement in bed-based CLP service provision. Open-ended responses were analysed thematically.

**Results:**

A total of 222 staff from diverse disciplines and settings participated. Most participants (65%) reported that patients were reviewed within an acceptable timeframe, and 90% were at least moderately satisfied with CLP performance. Frequently endorsed areas for improvement were enhanced communication, increased resource allocation (staff, skill-mix, outpatient clinics), development of referral guidelines, and greater integration with medical teams. Non-CLP staff expressed frustrations with barriers to service delivery, particularly limited telephone access and medical clearance hurdles. Multiple CLP respondents believed that CLP’s role continues to be poorly understood by non-psychiatry staff. They also called for digitally integrated workflows to improve efficiency.

**Conclusion:**

This study highlights the essential role of CLP services in Australian public hospitals and identifies workforce, leadership, and infrastructure improvements as key priorities for enhancing service quality.

## Introduction

Consultation-liaison psychiatry (CLP) services provide specialist psychiatric assessment and advice for patients with medical or surgical conditions, requiring close collaboration with staff across hospital disciplines.^1^ CLP involvement in patient care is associated with improved clinical outcomes, greater cost-efficiency, and shorter hospital stays.^2–4^ Maximising these benefits requires CLP services to be performing optimally.

Recent work has focused on developing reliable measures to guide CLP quality improvement. Common indicators include staffing levels,^5,6^ timeliness of response,^7–12^ satisfaction scores,^4,8–10,12,13^ and qualitative feedback.^4^ While self-evaluation promotes reflection and accountability,^7,13^ it is prone to bias, with clinicians potentially over- or under-estimating their impact.^12^ Accordingly, robust evaluation is best achieved by combining quantitative and qualitative methods,^12,14,15^ and obtaining perspectives from the range of hospital staff involved in patient care.^4,8^ This inclusive approach aligns with national and international guidelines^12,14^ and elicits more holistic and detailed insights into CLP service strengths and weaknesses.

Most Australian CLP evaluations have been single-site studies focused on referrer feedback,^7,9,10,12,13,16,17^ thereby limiting perspectives on performance-related issues common to Australian CLP services. However, multi-site evaluation may enable a broader understanding of areas of alignment and divergence between CLP providers and hospital staff, supporting a more unified view of quality improvement priorities in CLP services.

## Aims

This study aimed to identify key priorities for quality improvement in Australian CLP services through diverse perspectives obtained from CLP service providers and general hospital staff.

## Methods

### Design and setting

The study employed a cross-sectional survey of whole-of-hospital, bed-based CLP provision in Australian public general hospitals with active CLP services, which were identified via Department of Health websites. Institutional ethics committee approvals were obtained prior to commencement of the study.

### Participants and recruitment

Electronic survey invitations were emailed to CLP clinicians/managers, inpatient AOD liaison services, directors of advanced training in CLP, clinical directors of public mental health services, and managers of lived-experience, social work, and liaison clinical psychology teams. Invitations were also circulated via the RANZCP Psyche newsletter and private social media groups of CLP psychiatrists and nurses. Referring medical staff members were recruited at one metropolitan Tasmanian hospital and one major regional Victorian hospital via emailed invitations and QR-coded posters. Both hospitals had established CLP services with electronic referral systems and ≥2 psychiatric trainees, ≥2 consultant psychiatrists, and ≥1 CLP nurse.

### Eligibility criteria

Inclusion required employment in an Australian public hospital with an active CLP service and either providing CLP or history of referring to CLP. Small honoraria were offered to participants to increase the participation rate. The survey ran from August 2023 to February 2024.

### Data collection

REDCap software collected anonymised data on professional role, state/territory, hospital type, and number of prior CLP referrals. In line with recommendations for evaluation of CLP services,^12,14^ the survey combined ordinal questions with qualitative items to provide more meaningful information. The survey questions were as follows:

1. Does your CLP service review patients within an acceptable time frame? (5-point Likert scale ranging from ‘Never’ to ‘Always’).
2. How satisfied are you with the performance of your CLP service? (5-point Likert scale ranging from ‘Not at all satisfied’ to ‘Completely satisfied’).
3. How could your hospital’s CLP service be improved? (free-text response).

### Data and statistical analysis

Respondents were classified as (1) CLP providers (multidisciplinary CLP staff, liaison subspecialities, mental health clinical directors) or (2) non-CLP hospital staff. Likert data were summarised with frequency distributions and IQRs. An a priori sample size calculation (using data from Kovacs, et al.^8^; power 0.80) indicated minimum samples of 60 (satisfaction) and 98 (timeliness) to detect significant group differences. Groups were compared using Mann–Whitney U tests (SPSS v30; α = 0.05). Free-text responses underwent mixed-methods thematic analysis in NVivo v15. Authors MT and ZF independently coded sections of text into subthemes and themes. Cross-checking, reflective discussions, and multiple iterations were performed to increase reliability of the thematic results.

## Results

### Descriptive characteristics of the respondents

A total of 222 participants completed the survey, representing all targeted hospital types (regional *n* = 122, 55%; metropolitan *n* = 91, 41%; rural *n* = 9, 4%) and every Australian state and territory (Victoria *n* = 129, 58.1%; Tasmania *n* = 30, 13.5%; New South Wales *n* = 26, 11.7%; Queensland *n* = 16, 7.2%; South Australia *n* = 7, 3.2%; Western Australia *n* = 6, 2.7%; ACT *n* = 5, 2.3%; Northern Territory *n* = 3, 1.4%). The combination of state/territory and hospital type indicated representation from at least 17 distinct care settings.

Among CLP service providers (*n* = 120), participation was highest among psychiatrists (*n* = 42, 35%), nurses (*n* = 28, 23.3%), and registrars/trainees (*n* = 27, 22.5%) compared to other professionals (Clinical Director/manager *n* = 10, 8.3%; AOD liaison clinicians *n* = 8, 6.6%; CLP psychologist *n* = 4, 3.3%).

Among non-CLP respondents (*n* = 102), most were medical staff representing a range of seniority from medical and surgical specialities (Table 1). They were generally experienced referrers to CLP (median and modal values >20 previous referrals).

**Table 1.** Profile of the non-CLP hospital staff by role, speciality, and referral frequency.

Referrer characteristics	*N* (% of group)
Professional role	
Specialist doctor (consultant)	28 (27.5)
Registrar doctor	18 (17.6)
Intern or resident doctor	27 (26.5)
Liaison clinical psychologist	4 (3.9)
Social worker	11 (10.8)
Pharmacist	4 (3.9)
Peer support/lived experience worker	3 (2.9)
Other	7 (6.9)
Speciality (consultants and registrars)	
Acute and emergency	7 (15.2)
General medicine	10 (21.7)
Physician subspecialities^a^	12 (26.1)
Neurology	5 (10.9)
Surgical and procedural	5 (10.9)
Chronic and supportive care	4 (8.7)
Paediatrics	3 (6.5)
Number of referrals to CLP	
1	7 (6.9)
2–5	26 (25.5)
6–10	10 (9.8)
11–20	15 (14.7)
Greater than 20	44 (43.1)

^a^Multiple consultant physicians reported performing general medicine ward service alongside their routine specialist work but chose to identify themselves as a specialist physician in the survey. Acute and emergency = intensive care, perioperative medicine, toxicology, emergency medicine; general medicine and subspecialities = general medicine, cardiology, endocrinology, nephrology, gastroenterology, infectious disease, haematology, medical oncology, geriatrics; chronic and supportive = palliative care, rehabilitation, pain medicine; surgical and procedural = general surgery, orthopaedic surgery, and obstetrics and gynaecology.

### Does your CLP service review patients within an acceptable timeframe?

**Figure 1.**
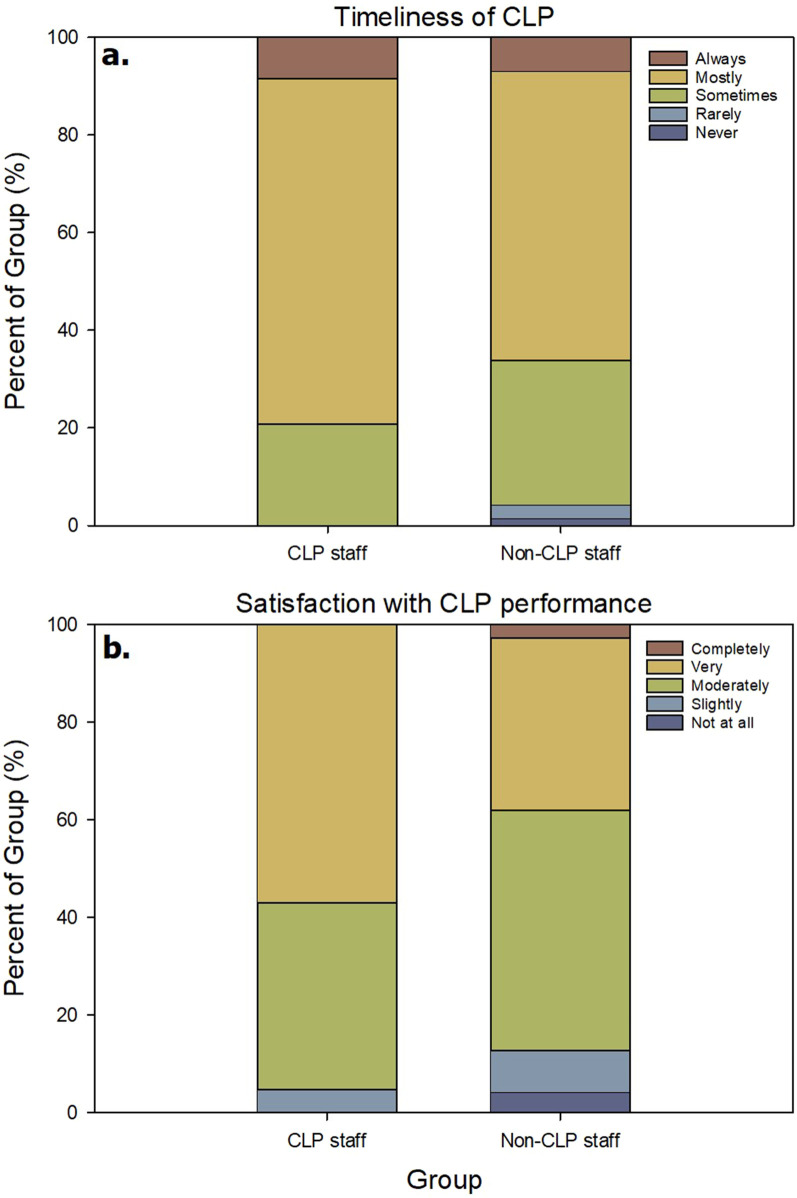
Distributions of responses from CLP staff and non-CLP staff about (a) how often CLP provides a timely response to referrals, and (b) the respondent’s overall satisfaction with performance of their CLP service.

A majority of participants (65%) reported that their CLP service mostly reviewed referred patients within an acceptable timeframe (IQR: sometimes–mostly). Only 3.3% indicated that timely reviews occurred rarely or never. CLP service providers more often selected mostly or always (mean rank = 52.9) than non-CLP hospital staff (mean rank = 46.3), although this difference was not statistically significant (U = 734, *p* = .24). The distribution of responses by group is shown in Figure 1(a).

### How satisfied are you with the performance of your CLP service?

Almost half of the participants (47.3%) reported being moderately satisfied with the performance of their CLP service (IQR: moderately satisfied–very satisfied). Overall, 90% were at least moderately satisfied, while 10% expressed dissatisfaction (not at all or slightly satisfied). CLP service providers reported higher satisfaction (mean rank = 53.7) than non-CLP hospital staff (mean rank = 44.4), which was not statistically significant (U = 595, *p* = .13). The distribution of responses across satisfaction categories for both groups is shown in Figure 1(b).

### How could your hospital’s CLP service be improved?

**Figure 2.**
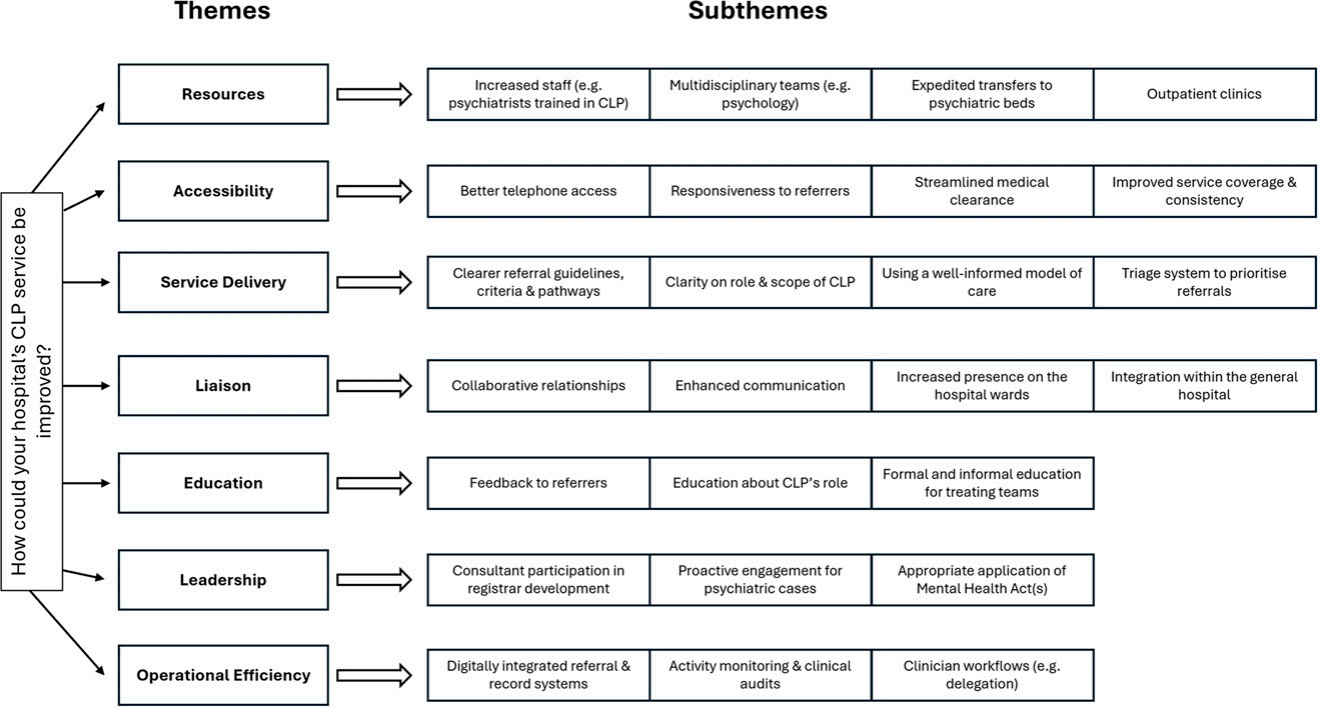
A flowchart of the themes and subthemes that emerged from respondent’s views about how their hospital’s CLP service could be improved.

Seven themes emerged from responses for improvement of CLP services (Figure 2). The most frequently identified areas were increased staffing (14% of codes), clearer referral guidelines (11%), and stronger collaborative relationships (11%), which together accounted for nearly one-third of all coded responses.

#### CLP group

CLP respondents emphasised the need for greater staffing capacity and service clarity. Examples included increased consultant and registrar FTE fully dedicated to CLP rather than split across roles, broader multidisciplinary skill mix, and expansion of outpatient services (e.g. oncology and neurology). These changes were viewed as key to improving after-hours coverage, continuity of care, and liaison with treating teams.

Like all public mental health services, the service is under resourced. CLP is defensive and can be difficult to engage in receiving referrals due to feeling work overload (CLP psychologist, VIC).

Participants identified the need for clearer service models, including defined scope of care, referral guidelines and criteria, simplified access pathways, and better integration with medical teams and subspecialities (e.g. addiction and neuropsychiatry). Suggestions to strengthen liaison included embedding trainees in medical teams to improve communication, proactively identify appropriate referrals, model assessment, and promote informal collaboration.

We are trialling having an advanced trainee rotate around the general medical teams, spending a week to shadow them on rounds provide demonstrations of risk assessment and basic psych screening at the bedside, to improve referral quality (CLP psychiatrist, NSW).

Issues with liaison, communication, and collaboration appeared to be prominent for urgent referrals.

It would be preferable to be given a bit more notice for patients admitted with overdoses/attempted suicide (whether ICU or general wards) instead of waiting until they are about to be discharged from hospital. Often an expectation that they call and we run (CLP nurse, NT).

Education for non-psychiatry staff was viewed as essential to improving understanding of CLP’s role and upskilling teams to manage distress and basic psychiatric issues independently.

Ongoing education about the role and scope of the service, and about the approach to and management of various common clinical scenarios in the general hospital (CLP trainee, WA).

Some trainees expressed dissatisfaction with limited exposure to CLP-trained psychiatrists. Several respondents highlighted the need for stronger consultant leadership and engagement within the hospital.

Greater collaboration and investment in dialogue about the role of the service in complementing other hospital services (Clinical Director, VIC).

CLP respondents also emphasised the need for operational efficiency through improved digital systems, integrated electronic medical records, and data collection tools for activity monitoring.

Providing CL without having all the relevant information available on EMR at your fingertips in your office, makes the job much less efficient (CLP psychiatrist, NSW).

#### Non-CLP group

Non-CLP respondents often highlighted both structural and communication barriers. Common concerns were inadequate staffing, limited after-hours coverage, lack of outpatient clinics for patients with chronic disease, delays in transfers to psychiatric beds, and difficulty reaching CLP doctors for telephone discussions.

Ease of access to discussions with the CLP team - very frustrating when you send a referral and get no feedback as to when teams will come to review. Also being able to call and discuss patients with the CLP team could be improved (Endocrinology registrar, VIC).

Like the CLP group, non-CLP staff emphasised the need for clearer referral guidelines, education about CLP’s scope, and stronger support for junior doctors when making and learning from referrals.

Each referral warrants a response even if the referral does not result in an assessment as each request is a teaching moment for both sides (Pain Physician, VIC).

Some noted that many patients were not referred due to uncertainty about referral criteria, while others cited delays caused by requests for low-yield pre-referral investigations. General hospital liaison clinical psychologists reported duplicated referrals and called for improved communication and clearer delineation of roles between CLP and liaison psychology, as well as greater referrer education about which team is most appropriate.

Respondents also stressed the importance of mutually respectful and collaborative relationships supported by timely communication, a willingness to review patients, stepwise management plans, greater ward presence, and visible assistance during emergencies.

Please look externally to your referrers to clarify what problems we have with psychiatry. Most of us feel dumped on by psychiatry and find the barriers and care refusals exhausting. Please just come and see the patients (Toxicologist, NSW).

More integration with the medical teams. At our hospital they are part of the area mental health service and tend to operate in a more ‘expert’ stance rather than collaborating with the treating team (Resident, TAS).

Several physicians urged CLP to take a more proactive leadership role in managing psychiatric deterioration and to expedite transfers for medically stable patients.

Be more proactive, including willingness to promptly take over care of a patient for whom the primary presenting complaint turns out to be psychiatric (Neurologist, VIC).

Early-contact CLP models, involving prompt acknowledgement of referrals and structured prioritisation systems, were viewed as desirable.

An early contact service (same or next day), not a full assessment and recommendation, but a documented visit, acknowledging and triaging the referral which may avoid some lower acuity or inappropriate referrals (Haematologist, VIC).

A minority expressed concerns about CLP management plans recommending conditional use of Mental Health Acts for voluntary patients.

Often our hospital’s treating team decides not to put a patient under an AO because they’re ‘voluntary’, but tells us that if they try to leave we should. If a patient does not have capacity and would hypothetically be stopped from leaving, they should be put under an AO from the start if that’s felt necessary (Resident, TAS).

## Discussion

The experiences of hospital staff with CLP services are an important indicator of service quality.^4,8–10,12,13,15–17^ This study gathered feedback from multidisciplinary staff across at least 17 Australian public hospitals, representing diverse professional backgrounds and experience levels. The absence of significant differences in timeliness and satisfaction ratings suggests that CLP and non-CLP staff held broadly similar overall perceptions of service performance. While overall satisfaction with CLP services was moderate-to-high, respondents also identified multiple key areas for improvement. This supports the view that mixed measures are required to evaluate CLP services.^4^ The quality improvement themes and subthemes offered meaningful insights into multiple challenges facing Australian CLP services. Overall, our findings provide a practical reference point for CLP teams to critically evaluate their service activity alongside established guidelines and benchmarks in the field.^4,14^

The most frequently identified area for improvement was inadequate CLP staffing. This finding aligns with recent national surveys of CLP psychiatrists and service directors in Australia^5^ and New Zealand,^6^ which also identified limited after-hours coverage, inadequate multidisciplinary expertise, underdeveloped liaison activity, and a lack of CLP outpatient clinics. These issues were similarly raised by respondents in the present study. Workforce shortages are associated with reactive compared to proactive care, reduced quality of care, missed KPIs, and unrecognised psychiatric comorbidities in general hospital inpatients.^18^ Addressing workforce deficits should thus be a strategic priority for Australian CLP services.

Our findings also mirrored issues identified in single-site Australian studies (QLD, VIC, NSW, WA), including referral difficulties,^9,10,12^ limited accessibility,^17^ consultation delays,^9^ communication and collaboration problems,^10,13^ role ambiguity of CLP,^16^ and inadequate education for non-CLP staff.^9,10,12^ Similar to our results, such issues can be problematic despite overall high satisfaction among referrers.^12,13,16,17^ Unique to the present study were frustrations related to medical clearance barriers, limited visibility or integration of CLP, lack of consultant leadership, and fragmented/non-digital clinical information systems. While these issues have been variably highlighted in previous evaluations of international CLP services,^8,15,19,20^ these problems being newly uncovered in the Australian setting may reflect a broader evaluation compared to previous studies or recent pressures on the Australian mental health system.

Non-CLP hospital staff expressed a desire for CLP services to be more available, facilitative, visible, responsive, proactive, and streamlined. Improved telephone access was viewed as critical for clarifying urgency and coordinating care. While CLP staff similarly expressed the need for improved communication and collaboration with hospital staff, they also emphasised improved education of hospital staff for capacity building in mental health assessment and management, improving understanding of CLP’s role, and digitally integrated clinical systems. Effectively, these changes would improve CLP’s workload and efficiency. Interestingly, there was no mention of the need for CLP telehealth outreach services^21^ or of strengthening scholarly development within CLP services, the latter being included in the CanMEDS framework for doctors and RCP PLAN standards.^14^ Assuming both areas are underdeveloped, their absence from responses may reflect overshadowing by more acute, visible service pressures.

Two-thirds of respondents reported that their CLP service mostly reviewed patients within an acceptable timeframe. This result warrants consideration given the benchmark that 90% of referrals should be reviewed by CLP within 24 h.^7,12,14^ Wood and Wand^12^ found that 91.5% of referrals were seen on the same day, corresponding with 93% referrer agreement that reviews were timely, suggesting a positive correlation between actual response times and perceived timeliness. Although the present study did not collect quantitative data on response times, the findings suggest that dissatisfaction was experienced by both CLP and non-CLP respondents for urgent requests (e.g. suicidal ICU patients and planned discharge following initial CLP review). When CLP services cannot accommodate highly time-sensitive requests, this can have a strong negative impact on perceptions of the service.^15,19,22^

Strengths of this study include strong participation and broad representation across disciplines and geographical regions, with participation rates closely reflecting typical speciality referral patterns.^23^ Limitations include overrepresentation from Victoria, Tasmania, and metropolitan or regional centres, as well as the exclusion of nursing staff and patients, which may have omitted important perspectives. Although input was sought from lived experience workers as proxies for patient views, their limited participation (*n* = 3) likely reflects low engagement with CLP services, highlighting an area for further development. Another limitation is potential self-selection bias, as those choosing to participate may have held stronger views than non-respondents, thereby influencing the balance of perspectives. This may partly explain why 10% of respondents reported dissatisfaction with their CLP service.

## Conclusion

This study reinforces the integral and multifaceted role of CLP services within Australian public hospitals and highlights several key areas for service improvement identified by both CLP teams and hospital staff. Many challenges likely reflect a more reactive versus proactive style of service delivery, underpinned by limitations in resourcing and clinical leadership. Addressing workforce shortages and modernising CLP infrastructure should remain strategic priorities to support responsive, integrated, and high-quality CLP services.
